# Childhood malaria case incidence in Malawi between 2004 and 2017: spatio-temporal modelling of climate and non-climate factors

**DOI:** 10.1186/s12936-019-3097-z

**Published:** 2020-01-06

**Authors:** James Chirombo, Pietro Ceccato, Rachel Lowe, Dianne J Terlouw, Madeleine C Thomson, Austin Gumbo, Peter J Diggle, Jonathan M Read

**Affiliations:** 1 Centre for Health Informatics, Computing, and Statistics (CHICAS), Lancaster University Medical School, Lancaster, UK; 2 Malawi Liverpool Wellcome Trust Clinical Research Programme, Blantyre, Malawi; 3 College of Medicine, University of Malawi, Blantyre, Malawi; 4 International Research Institute for Climate and Society, New York, USA; 5 Centre on Climate Change and Planetary Health & Centre for Mathematical Modelling of Infectious Diseases, London School of Hygiene & Tropical Medicine, London, UK; 6 Barcelona Institute for Global Health, Barcelona, Spain; 7 Liverpool School of Tropical Medicine, Liverpool, UK; 8 National Malaria Control Programme, Ministry of Health, Lilongwe, Malawi

**Keywords:** Malaria, Climate, Statistical model, Spatio-temporal, Vectors

## Abstract

**Background:**

Malaria transmission is influenced by a complex interplay of factors including climate, socio-economic, environmental factors and interventions. Malaria control efforts across Africa have shown a mixed impact. Climate driven factors may play an increasing role with climate change. Efforts to strengthen routine facility-based monthly malaria data collection across Africa create an increasingly valuable data source to interpret burden trends and monitor control programme progress. A better understanding of the association with other climatic and non-climatic drivers of malaria incidence over time and space may help guide and interpret the impact of interventions.

**Methods:**

Routine monthly paediatric outpatient clinical malaria case data were compiled from 27 districts in Malawi between 2004 and 2017, and analysed in combination with data on climatic, environmental, socio-economic and interventional factors and district level population estimates. A spatio-temporal generalized linear mixed model was fitted using Bayesian inference, in order to quantify the strength of association of the various risk factors with district-level variation in clinical malaria rates in Malawi, and visualized using maps.

**Results:**

Between 2004 and 2017 reported childhood clinical malaria case rates showed a slight increase, from 50 to 53 cases per 1000 population, with considerable variation across the country between climatic zones. Climatic and environmental factors, including average monthly air temperature and rainfall anomalies, normalized difference vegetative index (NDVI) and RDT use for diagnosis showed a significant relationship with malaria incidence. Temperature in the current month and in each of the 3 months prior showed a significant relationship with the disease incidence unlike rainfall anomaly which was associated with malaria incidence at only three months prior. Estimated risk maps show relatively high risk along the lake and Shire valley regions of Malawi.

**Conclusion:**

The modelling approach can identify locations likely to have unusually high or low risk of malaria incidence across Malawi, and distinguishes between contributions to risk that can be explained by measured risk-factors and unexplained residual spatial variation. Also, spatial statistical methods applied to readily available routine data provides an alternative information source that can supplement survey data in policy development and implementation to direct surveillance and intervention efforts.

## Background

While malaria has declined across Africa, analyses exploring the impact of nationally implemented control interventions have shown a mixed impact, with a recent analysis of malaria prevalence data across Africa since 1900 showing a complex range of driving factors including climate, socio-economic and environmental factors that may all depend on time and local context [1]. Climate affects many aspects of the transmission dynamics of malaria by its effects on the vector biology [2–6], and is expected to play an increasing role with progressive climate change. While global malaria control progress is monitored through malaria prevalence estimates from household surveys, national programmes in endemic countries often use facility based data to set impact targets and monitor progress, as this data is available on an ongoing basis and relates to disease burden rather than transmission. With efforts to strengthen the quality of routine facility-based monthly malaria data collection across Africa and progress in analytical methods to analyse collated data from multiple sources, this becomes an increasingly important data source.

Vector population larvae development depends on sufficient rainfall, yet excess rainfall can reduce numbers due to excessive water flow [7]. Temperature also plays a crucial role as the main vectors, such as tropical *Anopheles* mosquitoes, require temperatures between 16 and 32 °C to complete their life cycles. At higher and lower temperatures, there is high mosquito mortality [8]. Consequently, malaria displays seasonal patterns in response to changing climatic conditions.

In addition to climate, socio-economic factors play a critical role in malaria transmission [9, 10]. Therefore, addressing malaria through the design of optimal interventions can benefit from a clear understanding of the impact of both climate and non-climate factors.

The relationship between climatic factors and malaria incidence in sub-Saharan Africa has been extensively investigated. There is a general observation that as temperature increases in most parts of Africa, malaria incidence is projected to substantially increase [11]. Furthermore, the other non-climatic factors such as gross domestic product (GDP), malaria interventions will alter the malaria risk landscape [11]. In a study in Ethiopia, lagged effects of rainfall and temperature were found to be associated with malaria epidemics [12]. Rainfall and temperature lag of two months has also been found to be positively associated with malaria in South Africa [13]. Climate was found to be the leading driver of inter-annual variation in malaria incidence in Zimbabwe. In particular, rainfall, temperature and water vapour were found to be important predictors of increased malaria incidence [14]. A similar study in Mozambique found an association between malaria and relative humidity, rainfall and temperature [15].

Use of climate data to improve understanding of the observed trends and patterns in climate-sensitive diseases has not been widely undertaken in many African countries due to incomplete or unreliable climate and disease incidence data. The use of climate data derived from remote sensing provides an opportunity to investigate the impact of climate on malaria, even for areas where climate data from weather stations are sparse or non-existent.

Statistical models for aggregate and point-level data have been used to improve understanding of the interactions between vector-borne diseases (VBD) and environmental conditions [16–18]. Furthermore, work has been carried out towards the development of early warning systems for VBD such as malaria and dengue [17, 19–22]. In many settings, however, non-climatic conditions also play a key role in driving VBD and these act as confounding factors [23–25]. A purely climate-based model may thus not be sufficient to capture the complex relationships between VBD and the total environmental in general [26].

The roles of climate, geographic and socio-economic factors on malaria in Malawi were previously explored and disease incidence mapped covering the period 2004–2011 [27]. Since then, national control efforts have scaled up substantially, including the successful scale up of effective artemisinin-based combination therapy (ACT) since 2009, of malaria rapid diagnostic tests (RDTs) since 2011, and use of regular national net distribution campaigns since 2012 to move towards universal net coverage. Overall, malaria prevalence in children below 5 years of age has declined from 43% in 2010 to 24% in 2017 [28, 29].

This study aims to add to the evidence on the linkages between climate and malaria in Malawi and shows how the contribution of relevant non-climatic confounding factors can be visualized in a way that may help inform national malaria control programmes on options to take those factors into account and mitigate the impact of climate change. Using age-stratified malaria data from Malawi with climatic and non-climatic covariates, a spatio-temporal statistical model implemented in a Bayesian inferential framework was built and mapped explained and unexplained components of the spatio-temporal variation in malaria incidence.

## Methods

### Malawi context

Malaria is endemic to Malawi but with spatially varying levels of transmission [30] across a varied geographical landscape, from lowlands to highlands. Lakeshore districts generally have higher malaria prevalence than other districts. The country is divided into 5 climatic zones by the government’s meteorology department across 28 districts. Districts along the lake are generally of low altitude and have high average temperatures with average elevation ranges from 500 m above sea level along the lake and Shire valley to over 1500 in the central areas. Rainfall across Malawi varies, with average annual precipitation around 2500 mm in highland areas and 700 mm in low-lying areas [31].

### Data sources

Data were obtained from a variety of sources and collated at the district level, as shown in Table 1. In this paper, Likoma district, an island in Lake Malawi was excluded to give a contiguous study region. The analyses focused on known determinants of malaria prevalence and clinical diseases.

**Table 1:** Data sources. Climate and non-climate data variables, their description and source

Data	Description	Spatial resolution	Temporal resolution	Source
Malaria cases	Total cases (confirmed and suspected) reported by health centres in each district	District	Monthly	HMIS
Rainfall	Rainfall estimates (mm/month)	1km grid	Monthly	CHIRPS
Min. temp	Temperature estimates ($^\circ$C)	1 km grid	Monthly	NOAA NCEP
Max. temp	Temperature estimates ($^\circ$C)	1 km grid	Monthly	NOAA NCEP
NDVI	NDVI estimates	1 km grid	Monthly	LandDAAC MODIS
Population	Population estimates	District	Yearly	NSO population projections
Literacy	Proportion of population aged five and above that can read and write in any language	District	Yearly	WMS
Urban	Proportion of the population that stay in urban centres	District	Yearly	WMS
Area	Total district area	District		Unpublished reports
Altitude	Height above seas level (m)			NSO

#### Malaria data

The previous database in [27] was extended by adding routine malaria data for the period 2012 to 2017. Reported district-level monthly counts of confirmed and suspected malaria cases for 162 months collected between July 2004 and December 2017, checked and cleaned by the National Malaria Control Programme (NMCP) for completeness and consistency were used. Case data are recorded on paper forms at a health facility within a district, then aggregated monthly at the facility level. Facility data are subsequently aggregated to the district level and entered into an electronic database, the District Health Information System (DHIS) [32]. Within the Health Management Information System (HMIS) facilities self-report. Completeness of reporting is defined as the percentage of facilities that submit reports within the required deadline [33]. With time, the completeness of the data reported in the HMIS has been steadily going up, now standing at over 90%.

Over this period, different malaria diagnosis approaches were used. Prior to 2011, there was no widespread use of rapid diagnostic tests (RDT) as the policy had not been adopted leading to a high proportion of unconfirmed malaria cases. Therefore, the clinical diagnosis of malaria was widely used before 2011 [34]. The use of RDT was adopted in 2011 leading to a marked improvement in the quality of the data as an increasing percentage of cases were now being confirmed [35, 36].

#### Climate data

Climate data were obtained from satellite-derived archives from the library hosted at the International Research Institute (IRI). Monthly rainfall anomaly values averaged at the district level were obtained from the climate hazards group infrared precipitation with station data (CHIRPS) [37]. Temperature anomalies were obtained from the National Oceanic and Atmospheric Administration national centres for environmental prediction (NOAA NCEP) [38]. This data is based on the CPC monthly global surface air temperature data set at 0.5° from 1948-present. Temperature and rainfall anomalies are calculated as deviations from the long-term mean values and are preferred measures by climatologists when looking at trends over time [39]. For example, positive rainfall anomaly values indicate higher rainfall than the baseline value. A key advantage of using anomaly values is to reduce the effect of characteristics such as location or elevation which affect the absolute values. Normalized difference vegetative index (NDVI) data were collected from the LandDAAC MODIS satellite at a resolution of 1 km [40]. For the model-fitting, all gridded data were averaged over spatial areas corresponding to the districts in Malawi.

#### Non-climate data

To account for possible confounding between climatic variables and malaria, different non-climate data sources which included ITN use, population density, literacy levels and urban dwelling were used. In Malawi, mass ITN distribution started around 2012. Instead, data on the proportions of households with at least one ITN from the Demographic and Health Surveys (DHS) were used [41]. This was a proxy for the proportion of children under 5 sleeping under mosquito nets. Population density values were obtained from census reports. Data on literacy levels were obtained from the welfare monitoring surveys.

### Statistical framework and model

To estimate the variation in disease risk, the *standardised morbidity ratio* (SMR) was modelled. This is the ratio of observed to expected malaria cases within a single spatial unit in a single time-period and provides an estimate of the disease risk. The expected cases in each district are calculated by multiplying the district population with the annual observed risk. The annual observed risk is given by the total number of cases across all districts over the entire time period divided by the total population over the same period. SMR greater than 1 at a given time period suggests an excess risk of malaria in a district. More details on calculation of the expected cases are provided in section 1 of Additional file 1.

To describe the spatial and spatio-temporal variations in disease incidence, a Poisson-log-linear mixed effects model was applied. Let $y_{st}$ be the observed counts in spatial unit $s=1,\ldots ,N$ and time $t=1,\ldots ,T$, and $e_{st}$ denote the expected number of disease cases; the expected cases are calculated using standardization methods to take account of demographic differences in the populations across the different spatial units but without taking into account the effects of hypothesized risk factors or residual spatio-temporal variation [42]. It was assumed that

1 $$\begin{aligned} Y_{st} |e_{st},R_{st} \sim Poisson(e_{st}R_{st}), \end{aligned}$$

where $R_{st}$ is the relative risk of disease in spatial unit *s* at time *t*. In the log-linear mixed model,

2 $$\begin{aligned} \log (R_{st}) = x_{st}^\prime \beta + U_{st} \end{aligned}$$

where $x_{st}$ is a vector of covariates (fixed effects) with associated regression parameter $\beta$ and the random effects $U_{st}$ follow a multivariate Normal distribution with zero mean vector and covariance matrix $V(\theta )$ structured to include spatial and temporal components of variation. The relative risk $R_{st}$ is thereby decomposed into the explained and unexplained risks, $\exp (x_{st}^\prime \beta )$ and $\exp (U_{st})$ respectively. The unexplained risk component captures residual variation after accounting for all the covariates in the model.

By incorporating random effects, the Poisson log-linear model is able to account for overdispersion due to spatial autocorrelation, unstructured heterogeneity or a mix of the two [43]. Therefore, the spatially structured, unstructured and interaction random effects in the model are able to account for this overdispersion.

#### Model framework for the Malawi malaria data

The specific model formulation for the Malawi malaria data has been described in an earlier paper [27]. In brief, the notation for the model defined by 1 and 2 was extended to distinguish between cases under and over 5 years of age. Let $Y_{jst}$ be the monthly malaria count for age group $j=(1,2)$ corresponding, respectively, to ages 5 or more and 0 to 4, district $s=1,\ldots ,m=27$ and time $t =1,\ldots ,n=162$ months. Similarly, let $e_{jst}$ be the corresponding expected malaria count.

With this extended notation, the relative risk is written as $R_{jst}=x_{st}^\prime \beta +U_{st}$, where

3 $$\begin{aligned} U_{st}=P_s+D_t+G_{st} \end{aligned}$$

In Eq. 3, the terms $P_s$, $D_t$ and $G_{st}$ denote purely spatial, purely temporal and residual spatio-temporal components of variation in risk, respectively. Following [44] and [45] it is assumed that the $G_{st}$ are mutually independent, $G_{st} \sim N(0,\tau _I^2)$, and that the spatial random effect, $P = (P_1,\ldots ,P_m)$ and the temporal random effect, $D=D_1,\ldots ,D_n$ form Gaussian Markov random fields [46]. Specifically, the model defines spatial neighbourhood relationships through a symmetric $m \times m$ matrix $\mathbf {W}$ with elements $w_{ij} = 1$ if the spatial units *i* and *j* are neighbours, and $w_{ij}= 0$ otherwise; *i* and *j* are specified to be neighbours if they share a common boundary. Similarly, temporal neighbourhood relationships are defined by a symmetric $n \times n$ matrix $\mathbf {V}$; following [45], $v_{ij}= 1$ if $|j - i| = 1$ and $v_{ij} = 0$ otherwise. Now, writing $P_{-s}$ for the $(m-1)$ element vector obtained by removing the *sth* element from *P*, and similarly $D_{-t}$ for the $(n-1)$-element vector obtained by removing the* t*-th element from *D* the model can be defined through its full conditional distributions,

4 $$\begin{aligned} P_s|P_{-s}& \sim {\mathrm{N}} \left( \frac{\rho _S\sum _{j=1}^m w_{sj}P_j}{\rho _S\sum _{j=1}^m w_{sj}+1-\rho _S},\frac{\tau _S^2}{\rho _S\sum _{j=1}^m w_{sj}+1-\rho _S}\right) \end{aligned}$$

5 $$\begin{aligned} D_t|D_{-t}& \sim {\mathrm{N}} \left( \frac{\rho _T\sum _{j=1}^n v_{tj}D_j}{\rho _T\sum _{j=1}^n v_{tj}+1-\rho _T},\frac{\tau _T^2}{\rho _T\sum _{j=1}^n v_{tj}+1-\rho _T}\right) \end{aligned}$$

Both the $P_s$ and $D_t$ are mean-centred such that $\sum _{s=1}^{m}P_s=\sum _{t=1}^{n}D_t=0$

The following diffuse prior specifications for the fixed effect parameters $\beta$ and the random effect parameters $\vartheta = (\tau _{S}^2, \tau _T^2,\tau _I^2,\rho _S,\rho _T)$ were used. Firstly, independent Normal priors, $\beta _i \sim N(0,1000) : i\ =\ 1,\ldots ,p$ for the elements of $\beta$ were specified. Secondly, for the variance components $\tau _{S}^2$, $\tau _T^2$, and $\tau _I^2$, independent inverse-Gamma priors, $\tau ^2 \sim IG(1,0.001)$ were specified. Finally, independent uniform priors, $\rho \sim U(0,1)$ were specified for the autocorrelation parameters $\rho _S$ and $\rho _T$.

#### Model fitting for malaria data in Malawi

To account for differences in malaria diagnostics over time, a binary variable (0 before adoption of RDTs, and 1 after adoption) was defined. Firstly, a non-spatial generalized linear model (GLM) was fitted to investigate the association between the outcome and different covariates whether climatic or non-climatic and find significant predictors to include in the generalized linear mixed model (GLMM). The following covariates were included in the GLM; rainfall and temperature anomalies and their 3 month lags, NDVI, population density, RDT use, literacy rates, ITN use. Significant variables were then included in the GLMM.

To fit the GLMM, Markov Chain Monte Carlo (MCMC) techniques were used to simulate from the posterior distribution using a combination of Gibbs and Metropolis-Hastings algorithms to estimate model parameters. Three chains of length 300,000 were generated with a burn-in of 50,000 iterations. Every fiftieth iteration was retained to obtain a sample of 5000 approximately independent realisations from the joint posterior distribution of $\beta$, $\theta$ and *U* for post-processing. The Geweke diagnostic test which is based on the *Z* test for equality of means was used to check convergence [47]. The Markov chain is partitioned into 2 disjoint segments and it tests whether the means in the two segments are equal. If the Markov chain has reached stationarity, the Geweke statistic asymptotically follows a standard normal distribution. More details on model fitting including convergence diagnostics are described in sections 3 and 5 of Addtional file 1. All analyses were performed in the R environment for statistical computing [48]. The models were fitted using the R package CARBayesST [45]. The R code for the analysis can be found in Additional file 1.

## Results

### Clinical malaria patterns

**Fig. 1:**
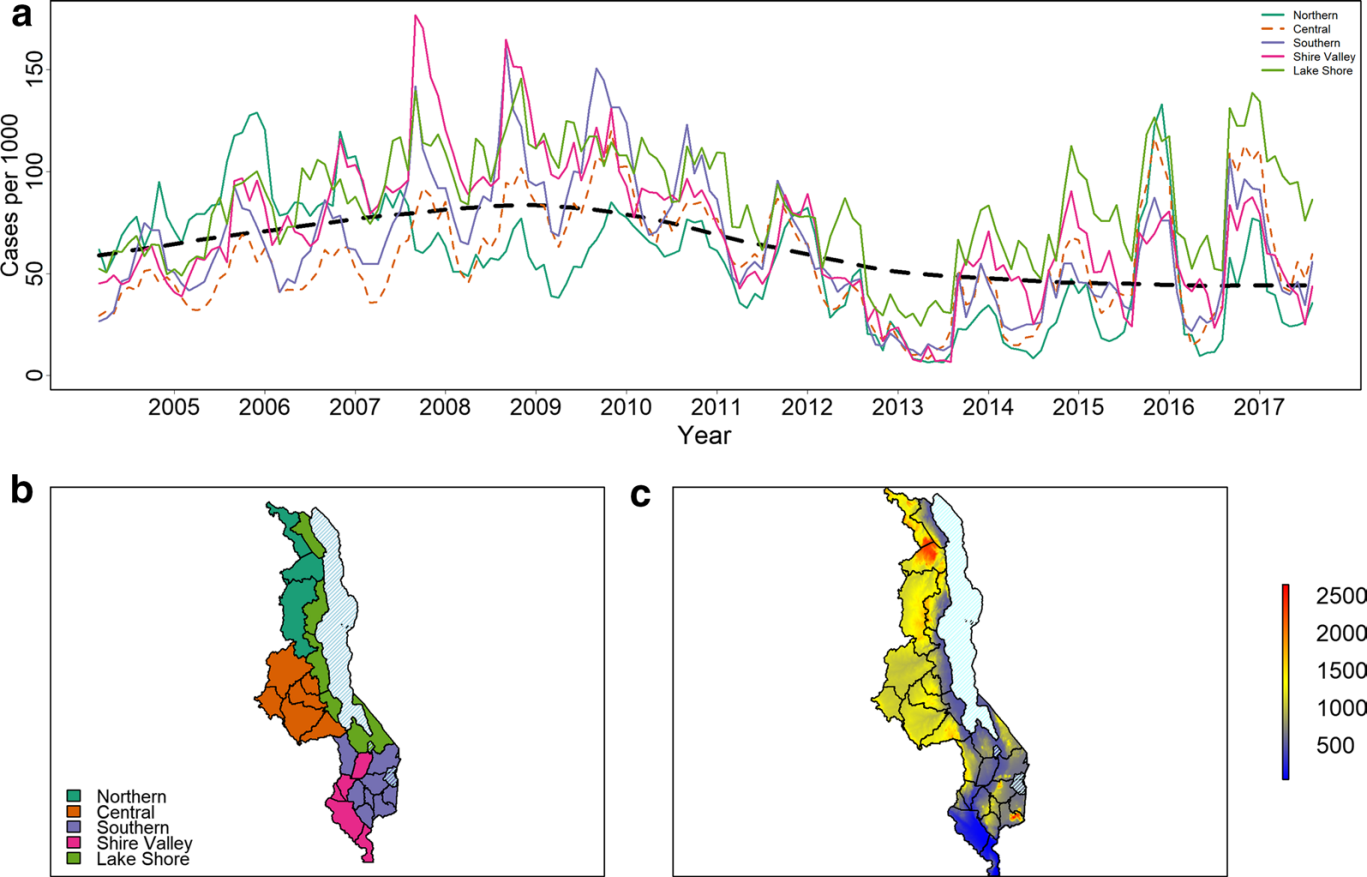
Annual under-five malaria burden from 2004–2017 by climatic zone and their location in Malawi and their relative altitude. **a** Temporal changes in under-five malaria by climatic zone. **b** Relative location of climatic zones within Malawi. **c** Underlying altitude of the climatic zones

The malaria case rates between 2004 and 2017 period are shown by climatic zone in Fig. 1. During this period the annual malaria incidence over time showed a decrease in incidence between 2009 and 2013 followed by a slight increase from 2014 to 2015. Overall, there is a general reduction in malaria rates over the 2004–2017 period as shown by the dotted smooth line.

**Fig. 2:**
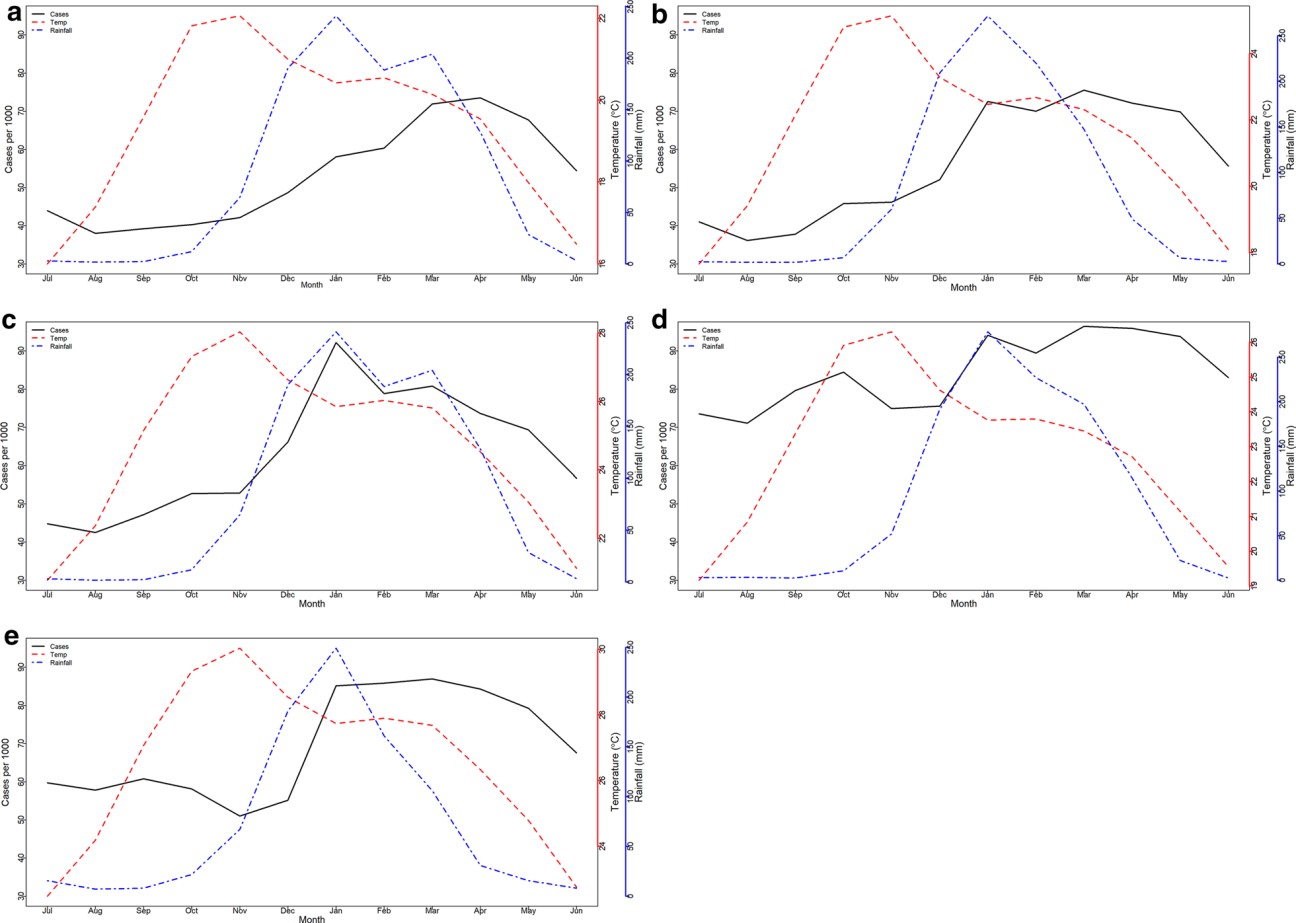
Relationship between monthly mean temperature, rainfall and malaria. Monthly average malaria incidence, rainfall and temperature at the climate zonal level. **a** Northern zone. **b** Central zone. **c** Southern zone. **d** Shire valley. **e** Lake shore. The red dotted line is the mean temperature while the blue dotted line is the mean rainfall. The disease incidence is shown by the black solid line

Detailed seasonal patterns of malaria case rates, rainfall and temperature are shown for each climate zone in Fig. 2. Across zones and geographical areas, there are similar patterns of seasonality. Peak temperatures occur between October and November, before the start of the rainy season. Rainfall peaks in January, with a lag period of 0 to 3 months of peaking malaria incidence.

**Fig. 3:**
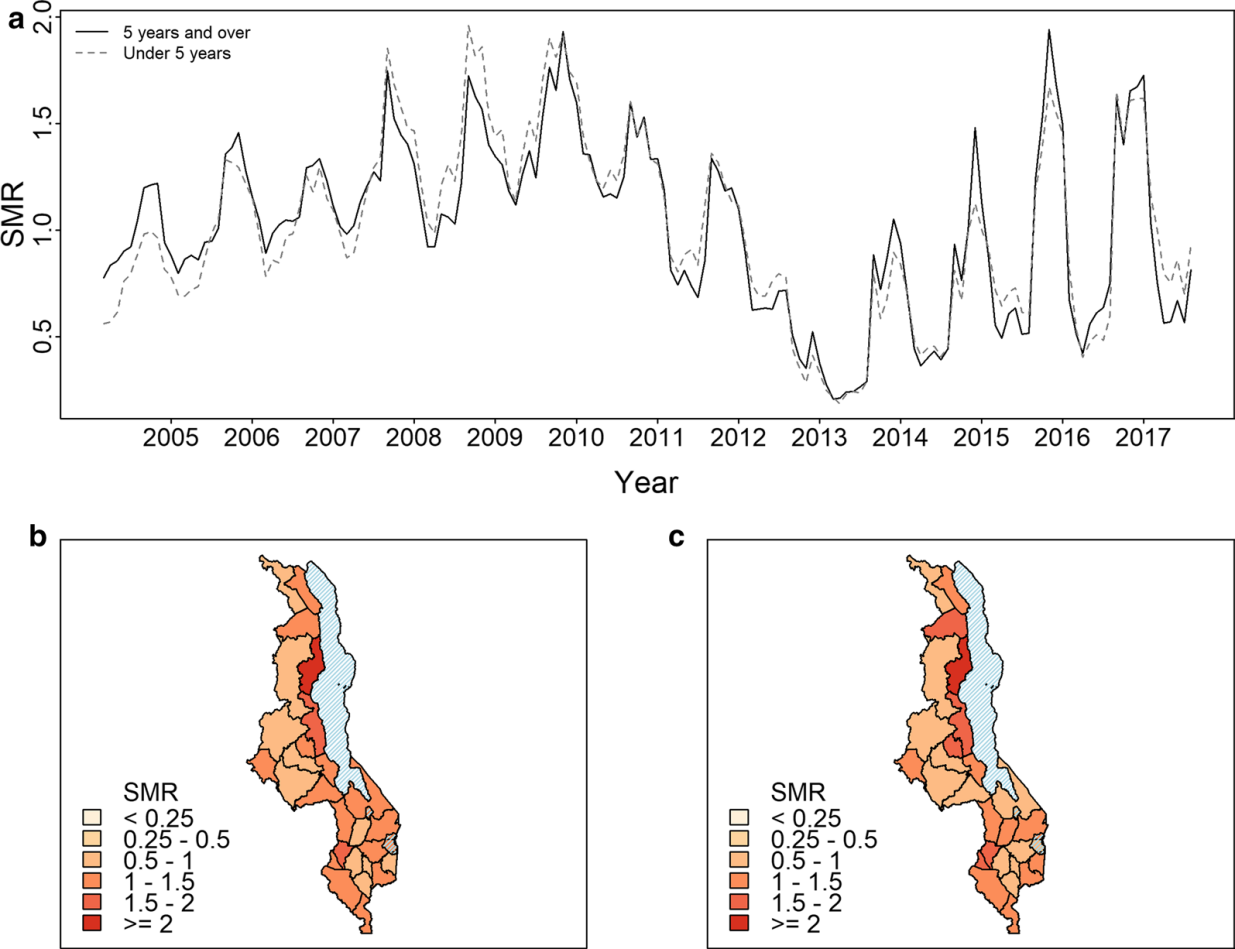
Malaria SMR averaged over time and space for the period July 2004–December 2015. Standardised morbidity ratio (SMR) for Malawi: **a** averaged across the country for each month, **b** averaged over time for each district for the age group 5 years and over, **c** averaged over time for each district for the under 5 years age group

Figure 3 shows the marginal spatial and temporal variations in malaria SMR across Malawi. The temporal variation (Fig. 3a) indicates similar patterns of seasonality and inter-annual variation in both age groups. The spatial variation in the SMR for the age group 5 years and under (Fig. 3b) shows higher malaria incidence in some of the districts along the lakeshore and Shire Valley regions. For the over-five age group (Fig. 3c), a similar pattern is observed.

### Model estimates

#### Association between climate and non-climate factors with clinical malaria

The following covariates were included in the GLMM: mean rainfall and temperature anomalies; rainfall anomalies lagged by 1 to 3 months, temperature anomalies lagged by 1 to 3 months, NDVI, literacy (as a proportion of the district population) and population density. An indicator variable to specify the time before and after adoption of RDTs was also included in the model.

In the spatio-temporally structured model, clinical malaria incidence was associated with rainfall at 3 month lag, temperature including all lags and NDVI. Relative risks and their 95% Bayesian credible intervals are shown in Table 2.

**Table 2:** Parameter estimates for the mixed model. Estimates for relative risk for climatic and non-climatic parameters respectively with associated 95% credible intervals

	RR	95% credible interval
Rainfall	1.00	(1.00, 100)
Rainfall lag 1	1.00	(1.00, 1.00)
Rainfall lag 2	1.00	(1.00, 1.00)
Rainfall lag 3	1.03	(1.01, 1.05)
Temperature	1.03	(1.00, 1.05)
Temperature lag 1	1.03	(1.00, 1.06)
Temperature lag 2	1.05	(1.03, 1.08)
Temperature lag 3	1.04	(1.01, 1.07)
NDVI	1.74	(1.45, 2.07)
Literacy	1.00	(1.00, 1.00)
Pop. density	1.00	(1.00, 1.00)
RDT	1.27	(0.96, 1.68)

After allowing for residual spatio-temporal dependence, rainfall was no longer statistically significant in the current month. However, there was a slight positive relationship between malaria incidence and rainfall in the three months prior. A unit increase in rainfall anomaly was associated with a 3% increase in malaria burden (RR = 1.03, CI 1.01, 1.05). For temperature anomalies in the current month, with every 1 °C increase, estimated malaria incidence increased by 3% (RR = 1.03, CI 1.00–1.05). Malaria was also associated with temperature anomalies at 1–3 month lags with increase in malaria of 3%, 5% and 4% respectively. NDVI was also positively associated with malaria incidence, i.e. an increase in vegetative cover is associated with a 74% increase in malaria incidence (RR = 1.74, CI 1.45–2.07). It was also observed that population density did not show an association with malaria (RR = 1.00, CI 0.99–1.00). For literacy, no association with malaria was not observed also (RR = 1.00, CI 1.00–1.00). Lastly, a 27% (RR = 1.27, CI 0.95–1.68) increase in incidence was observed in the post RDT adoption period compared to before. The Geweke diagnostic scores for each covariate indicated convergence with the test statistic falling between the cutoff points, $(-1.96,1.96)$.

#### Mapping explained and unexplained variation in SMR

**Fig. 4:**
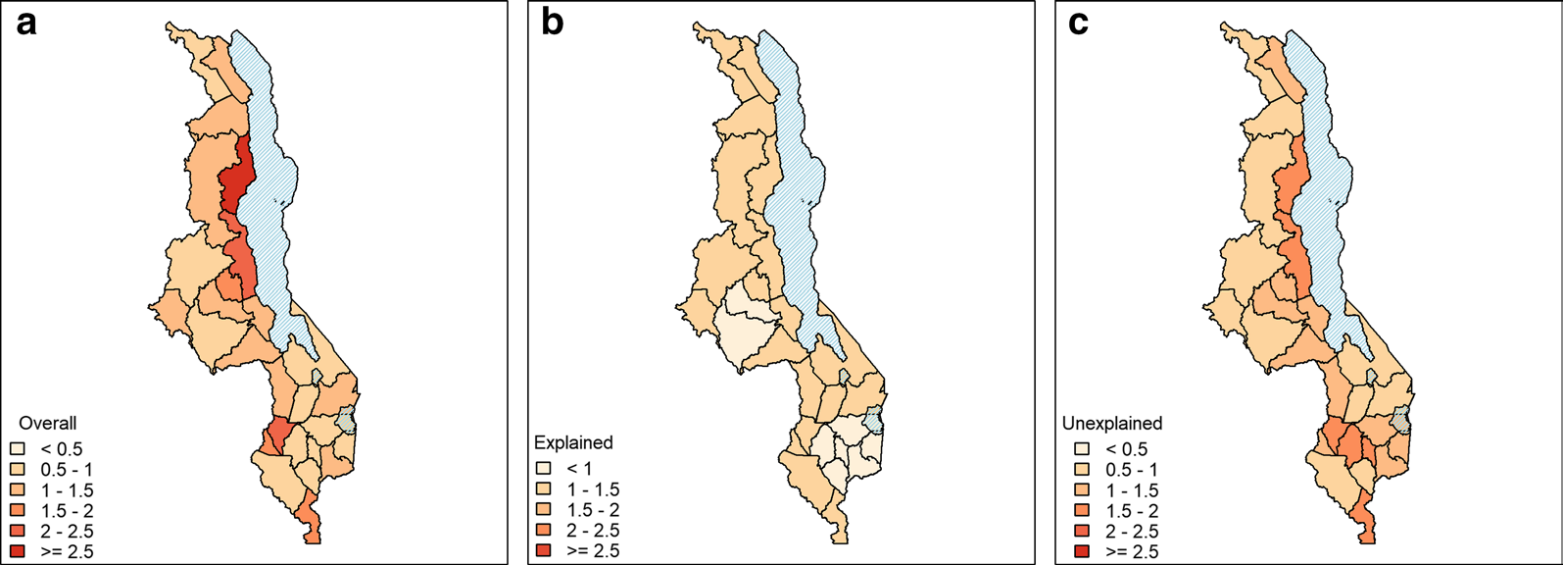
Contribution of various model components to the risk. Contributions to the overall malaria risk. **a** Overall risk $R_{st}$ due to combined effect of climatic, non-climatic covariates and non-observed covariates, **b** explained risk, $\exp (x_{st}^\prime \beta )$ due to observed climatic and non-climatic covariates, **c** unexplained risk, $\exp (U_{st})$ due to unobserved effects only

Figure 4 shows the decomposition of the overall risk into its explained and unexplained components. Fig. 4a shows the overall malaria risk, $R_{st}$ averaged over Malawi for the entire period. The final model predicts a higher than average risk in the lake shore and Shire Valley districts and climatic zones. In addition, 3 of the districts in the central zone also show an elevated malaria risk compared to other districts in the zone. Fig. 4b, c show the explained and unexplained component of spatial variation in risk respectively, again averaged over time. In terms of model performance, the unexplained variation $\exp (U_{st})$ is relatively high in some parts of the country indicating the presence of other district specific non-observed variables.

**Fig. 5:**
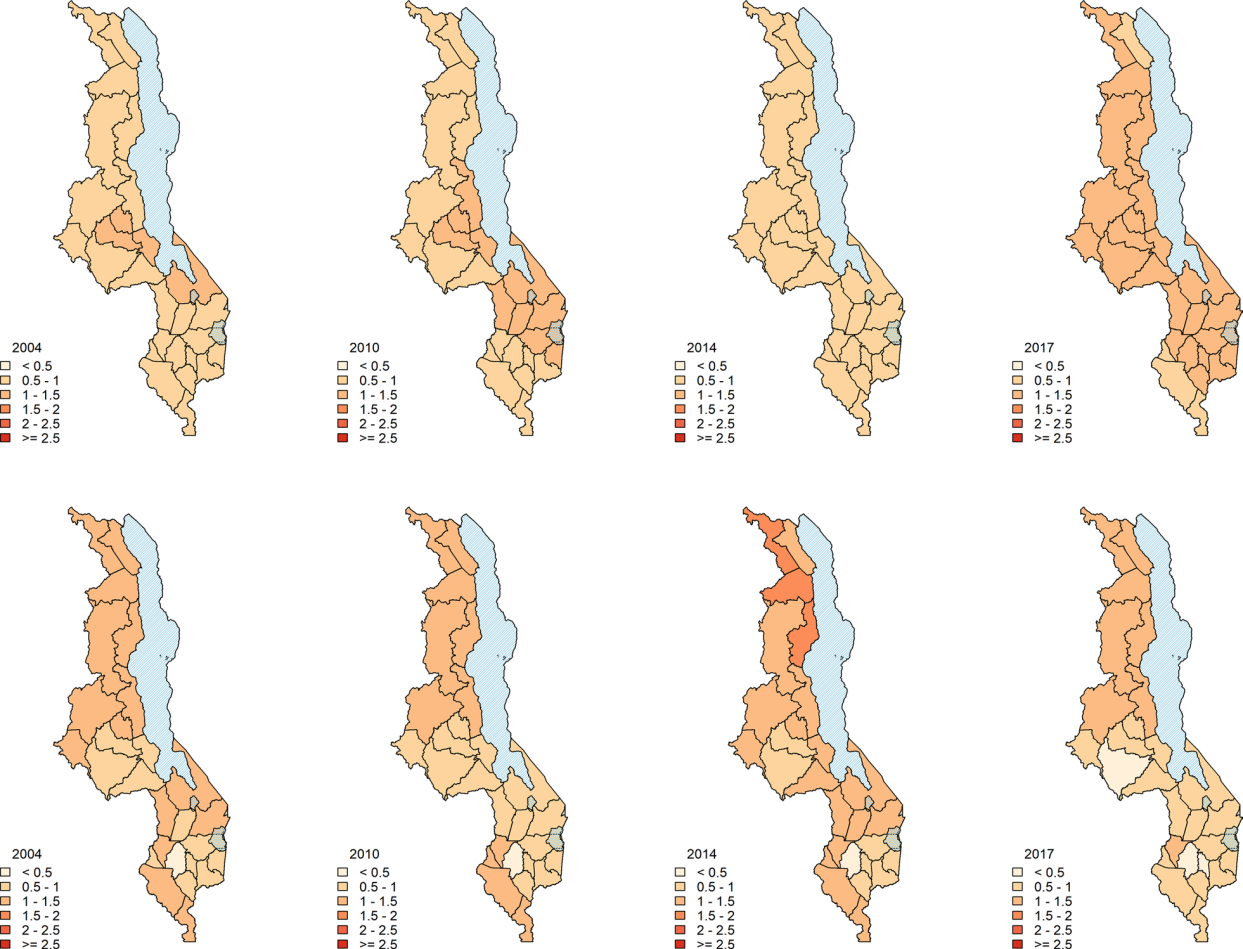
Contribution of model components to observed malaria risk over the study period. Contribution of climatic covariates (top panel) and non-climatic covariates (bottom panel) to malaria risk at different time points during the period from 2004 to 2017

Figure 5 shows the relative contribution of modelled climate and non-climate factors at four time points over the 13 year period. It is observed that the risk explained by modelled climate covariates (upper panel) does not show much variation between districts over time across the country. Temperature and rainfall three months prior were found to be significant. This shows that temperature and rainfall play a key and constant role in malaria transmission across the country. However, there is an observed increase in the observed risk towards the end of the period under study in 2016/17. Lastly, there is a more varied explained risk by non-climate covariates (lower panel). There is a generally higher explained risk in the northern areas and Shire river valley region. The year 2014, in particular, indicates a high risk in this region.

## Discussion

Results show the added benefit of including climate and non-climate information in modelling of malaria incidence data. Temperature and lagged rainfall were found to be significant drivers of malaria. A spatio-temporal statistical model was fitted to quantify the effect of different climate and non-climate covariates on malaria incidence and to predict the incidence over the period July 2004 to December 2017.

The information on the relationships between the lagged effects of temperature and rainfall will help in the timing of impactful interventions. The areas with elevated risk revealed by the model will allow for a targeted application of interventions while the relatively long period of time considered in this study provides a temporal aspect necessary to understand the changes in malaria burden over time. The impact of climate and non-climate factors, therefore, provides an important information source for the design of optimal interventions which can cover the most affected districts while concurrently being time relevant.

While the quality and suitability of routine facility-based data is often questioned, the reported malaria incidence trends in time and space, in terms of seasonality and climate zone, largely follow the expected patterns and align with previously published malaria prevalence maps [18, 27]. The approach discussed in this paper uses more recent data and covers a relatively longer time period hence similarities with the earlier maps. The lakeshore and Shire valley areas are generally low-lying areas with higher average temperatures and higher malaria incidence. Major urban centres of Blantyre and Lilongwe show a lower risk through out this period.

When using routine incidence data to monitor control impact and long-term trends, control programmes need to take into account intervention implementation, climate and non-climate covariates from different data sources to improve the analysis and interpretation of disease incidence patterns.

In this study, incidence showed a steady decline in paediatric and adult data from 2009 following the introduction and gradual scale up of efficacious artemisinin-based combination therapy (ACT) in 2008 and start of ITN distribution to mothers and children, this decline was followed by a nadir and upsurge after 2013. While similar reversals and increases have been reported by countries in the southern African region [49], their control programmes included different control implementation stages during this period, suggesting other factors could be at play. Analysing climate and non-climate factors, and visualizing the explained and unexplained components of the observed variation in disease risk, can give additional insight. While the introduction and scale up of malaria rapid diagnostic tests (mRDTs) and inclusion of community-based malaria treatment from 2012 could have led to increased health-care seeking behaviour and capture of cases that previously did not present to the health care system, the period after 2013 also documented higher average temperatures. In the modelling framework, even when ITN intervention were included in the spatial models, the recent rise in incidence could not be explained away. Thus neither climate changes nor changes in intervention intensity could account for incidence trends. More details are in section 6 of Additional file 1.

In terms of model performance, the unexplained variation is lower across the country. This shows that most of the variation has been captured by the covariates in the model. However, the substantial unexplained risk shows the importance of including random effects in the model.

The non-significance of rainfall on malaria in the current month shows the complexity of the relationship between malaria incidence and climate in general, but rainfall in particular. Studies in different settings have shown mixed effects of rainfall on malaria; some have shown a positive association, whilst others have shown a very weak or no association [50–52]. NDVI is also significantly associated with malaria. Seasonal and year to year changes in NDVI are commonly associated with rainfall. Green vegetative cover, which is prevalent in the rainy season, is positively associated with malaria incidence. Several other studies have shown vegetative cover to be a significant predictor [53, 54]. Temperature plays key role in the development of malaria vectors and their activities that directly or indirectly lead to the spread of malaria. It has been found to be a significant ecological factor in several studies such as [55–57] but its impact on malaria transmission in tropical climates is usually considered a highland phenomenon [58].

The findings, in general, follow a similar pattern of the impact of climatic factors on malaria. Though there may be differences between studies regarding which climatic factors (and their associated lags) are more importantly associated with malaria, the underlying role of climatic factors as a key driver of malaria incidence is shared between this study and many others. This knowledge is likely to be of key importance in future control efforts in the face of continuing climate variability across most of Africa.

Both components of the decomposed risk (overall and covariate-explained) could be affected by other important factors that were not considered in these analyses. This include the completeness and quality control of monthly reports from government and faith-based health facilities that do report into the DHIS2 system, but could also come from other health facilities, mainly private for-profit, that do not report their data to the MoH via the DHIS2. While data on reporting completeness or quality at district level was not accessed, it is likely that reporting rates influence the estimation of the malaria burden in Malawi.

While analyses show how climate and non-climate data from multiple sources can be used to improve the analysis and interpretation of routine malaria data patterns, there are some limitations and strengths of the Malawi data over the reporting period and potential steps moving forward.

Self-treatment at home will never be captured in the HMIS. Any substantial changes in the proportion of home-treatment within the country over time could affect routine facility-based disease trends. In Malawi, however, the availability of antimalarials in rural areas is limited and treatment is provided for free by the government. The introduction and scale up of RDTs in 2011 and the programmes and steps to link the reported diagnosis and treatment to consumables stock management over the past year, provide reassurance on the reported cases moving forward. Prior to 2011, when the MoH adopted the policy of testing all suspected cases by RDT, [35], clinical diagnosis of malaria was widespread in Malawi. This may have affected the accuracy of the reported cases in the period before 2011.

Selection bias in seeking health care due to differential access to health facilities among different groups of people and variable distances between facilities and homes is another common concern with routine facility-based data [59]. People living very far away from health facilities from may not be adequately represented in routine data. Actually, the inclusion of community-level diagnosis of malaria using mRDTs by community health workers in hard-to-reach areas in Malawi has been included in reporting to the DHIS2 since 2012 and may have contributed to the increase in reported cases across the country, but as they are included within the health facility level reports for the relevant catchment area, it was not possible to confirm this in the current analyses.

Satellite-derived climate data were used in the models, rather than directly measured climate data from weather stations. Ideally, a high-quality gridded climate database including rainfall and temperature (minimum and maximum) from weather stations should be used to formulate models and produce malaria risk predictions. Unfortunately, these data sources are not readily available in most developing countries due to a sparse network of weather stations. National climate data sets which integrate global products and all relevant local observations managed by the national meteorological agencies are increasingly available in African countries [60].

Intervention coverage status data was not available at district level for the period of interest, as this data is not part of the routine data collection and is assessed at regional level in the national household malaria indicator surveys. The presented model relied on crude intervention implementation proxies.

In terms of the modelling framework, there is a risk of ecological fallacy due to the differences in individual and aggregate associations in the outcome and predictor variables. The aggregation also presents a loss of information. Supplementing aggregate data with individual-level data is a solution to solving ecological fallacy. In the absence of individual data, the ecological analyses still provide useful information for understanding impacts at the aggregate level. Where the focus is on the prediction of aggregate-level outcomes, ecological fallacy, though present, may not be a big concern [61].

Despite these limitations, the presented work shows the potential added value of the spatio-temporal statistical modelling approach. Furthermore, there are three promising developments in Malawi that will soon offer opportunities to apply the framework with more detailed data on key covariates. First, as part of a collaboration with the LINK programme in Malawi [62] intervention coverage maps will soon become available for key interventions including ACT, mRDTs and ITNs, allowing integrating coverage scale-up. Secondly, as the LINK programme modelled spatio-temporal prevalence data at district level, there will be opportunity for more comparative analyses of modelled transmission and burden data. Lastly, electronic facility level reporting of clinical cases into the DHIS2 began in 2018, which will soon allow more granular mapping of disease risk at health facility catchment area, providing the opportunity to analyse more detailed spatial patterns moving forward. With these developments, the presented model framework can be expanded towards more in-depth analyses of intervention impact.

Furthermore, while acknowledging the severe consequences of climate change and climate variability on health outcomes and that the health sector has been slow to act compared to other sectors such as agriculture, the Ministry of Health (MOH) has been moving towards integrating climate change and health interventions into its programming by setting up a permanent climate change and health office, forging closer links with the department of climate change and meteorological services, and other agencies among other measures. The profile of climate change and human health programmatic area in Malawi has steadily been rising with funding from projects such as the World Health Organization (WHO) supported Global Framework for Climatic Services (GFCS) now being made available for specific interventions. The model outputs provide a resource that offers insight to programme managers on seasonality and disease burden that can help inform more targeted interventions. The GFCS project aims to encourage the integration of climate information for decision making in climate-sensitive sectors such as health. Therefore, this analysis fits well with the health sector’s strategic direction.

## Conclusion

This work provides a modelling framework for integrating climatic and non-climatic information into analyses of routine malaria case data at facility-level, in order to improve understanding of climate effects on climate-sensitive VBD such as malaria, while simultaneously controlling for non-climatic risk factors. The findings show the value of collaborations between control programmes, health researchers and climate experts in the collation, analyses and interpretation of routine malaria data. Visualizing the findings in maps produced provide easy to use tools for malaria control programmes to support their interpretation of disease trends over time, which, with the development of user friendly analysis tools could be incorporated into Technical Working Groups (TWGs) and standard programme review processes.

## Supplementary information

**Additional file 1.** Exploratory analysis, model fit and diagnostics.

## Data Availability

Malaria data from July 2011 was obtained from the DHIS and is freely available upon registration on http://live.hispmalawi.org.mw/. Data from July 2004 to June 2011 was obtained from the old non-web based DHIS system and was kindly provided by the MoH. The climate and environmental data can be obtained from IRI.

## Abbreviations

AIC: Akaike Information Criterion
ACT: artemisinin-based combination therapy
CAR: conditional autoregressive (model)
CHIRPS: Climate Hazards group Infrared Precipitation with Stations (data)
CPC: Climate Prediction Centre (temperature)
DHIS: District Health Information System
DHS: Demographic and Health Survey
GDP: gross domestic product
GFCS: Global Framework for Climatic Services
GLM: generalized linear model
GLMM: generalized linear mixed model
HMIS: Health Management Information Systems
IRI: International Research Institute
MCMC: Markov Chain Monte Carlo
MIS: malaria indicator survey
MOH: Ministry of Health
NDVI: normalized difference vegetative index
NOAA: National Oceanic and Atmospheric Administration
NSO: National Statistical Office
RDT: rapid diagnostic tests
SMR: standardized morbidity ratio
TWG: Technical Working Group
VBD: Vector Borne Diseases
WHO: World Health Organisation
WMS: welfare monitoring survey
