# Supplementary material for: Childhood malaria case incidence in Malawi between 2004 and 2017: spatio-temporal modelling of climate and non-climate factors
Source: Malar J. 2020 Jan 6;19:5. doi: 10.1186/s12936-019-3097-z (PMC6945411; doi:10.1186/s12936-019-3097-z)
Supplement: Supplementary file 1 — Additional file 1. Exploratory analysis, model fit and diagnostics. [file 12936_2019_3097_MOESM1_ESM.pdf]

Additional file: Exploratory analysis, model fit and diagnostics

1 Calculation of expected cases and SMR

The expected malaria case-counts e_{st} were calculated by multiplying the overall malaria risk for Malawi, π and the population of each district p_{st} , i.e. $e_{st} = p_{st}\pi$. The overall malaria risk for Malawi is given by dividing the total number of cases in Malawi with the total population, i,e, $\pi = \frac{\sum y_{st}}{\sum p_{st}}$ where y_{st} is the observed malaria counts in district s at time t and p_{st} is the corresponding population. The logarithm of the expected malaria counts is then included in the model as offset with a coefficient of 1 and hence no effect on the response variable. The standardised morbidity ration (SMR) is given by the ratio of observed estimated cases, i.e. $SMR = \frac{y_{st}}{e_{st}}$. The maximum likelihood estimate (MLE) of the risk R_{st} of a district in a GLM without random effects is the corresponding SMR, i.e. $R_{st} = \frac{y_{st}}{e_{st}}$. In the mixed model setting, the posterior mean of the relative risk for each district is therefore a weighted average of the SMR for the district and the prior mean of the relative risk in the overall spatial region giving rise to smoother estimates than those provided by raw SMR estimates.

2 Exploratory analysis

To provide a more detailed description of the national malaria incidence pattern, Figure S1 shows a decomposition of the monthly incidence series into seasonal, trend and residual components using the loess method (Cleveland, Cleveland, McRae, & Terpenning, 1990).

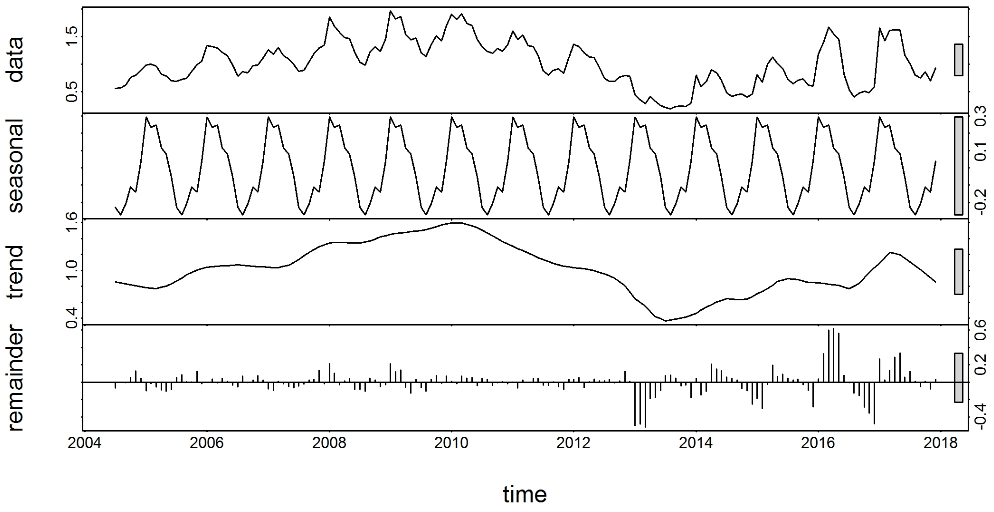

Figure S1: Decomposition of the malaria time series from July 2004 to December 2015 into the different components.: (A) observed incidence, (B) seasonal pattern, (C) observed trend and (D) random noise.

The trend component in Figure S1 shows a steady rise between 2004 and 2010, followed by a sharper drop between 2010 and 2013, and another rise since 2013. Both climatic and non-climatic factors could affect this. The period 2010 to 2013 coincided with a number of national-level policy interventions, whilst reporting mechanisms over time could also induce spurious trends; see below for further discussion.

The overall reporting rate from all the health facilities in the Malawi health system has been rising steadily over the years (Ministry of Health (MOH), 2015). Districts with better infrastructure are more likely to deliver better quality data. In general, data quality varies between districts.

Within the climatic zones, districts also show different patterns of malaria incidence. Climate is likely to be responsible, in part, for the observed differences in the incidence due to variable climatic conditions between zones. Figure S2 shows monthly malaria incidence in selected districts from each of the climatic zones. From the figure, it is apparent that malaria is endemic to all regions and zones and that there is inter-annual variation in the incidence. The problem of

low data quality, partly caused by a gap in reporting is highlighted in one of the districts.

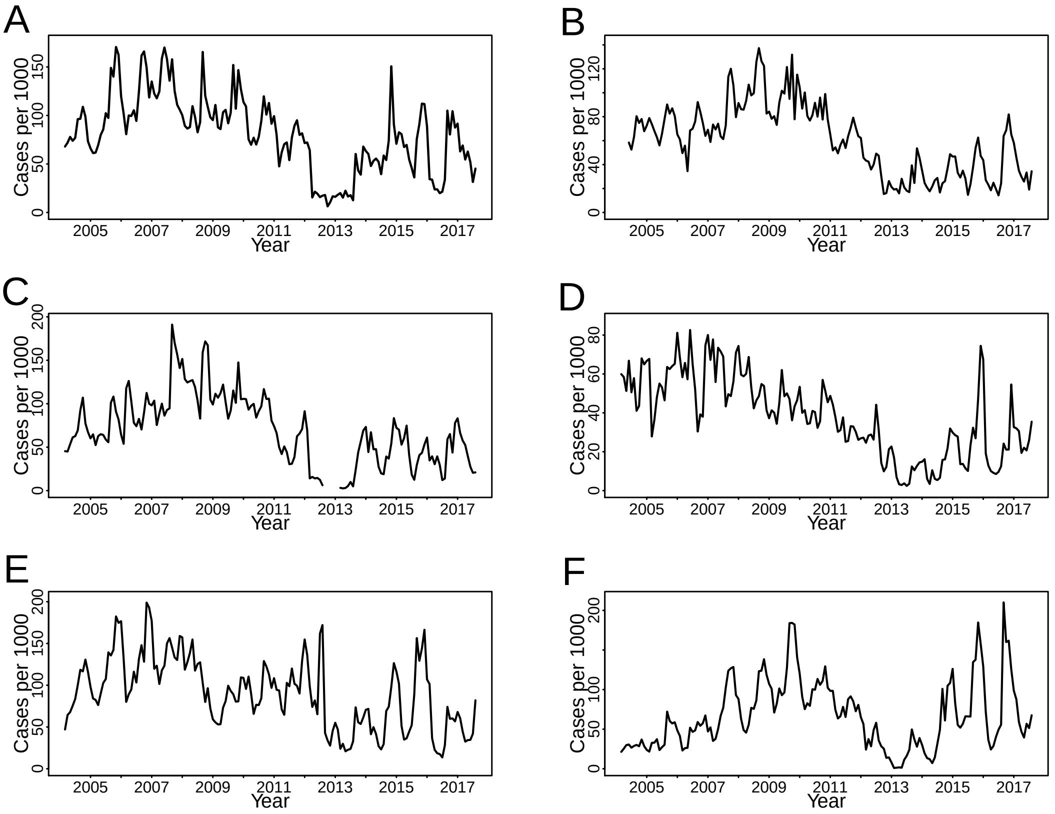

Figure S2: Malaria incidence in districts selected from each climatic zone covering the period July 2004 to Dec 2017 showing the seasonality patterns in malaria (A) Mangochi (B) Blantyre (C) Chikwawa (D) Chitipa (E) Rumphi (F) Dedza

3 Model fitting

We use a two-stage strategy to fit a general model within the class defined by equations

$$Y_{st}|e_{st}, R_{st} \sim \text{Poisson}(e_{st}R_{st}) \tag{1}$$

$$\log(R_{st}) = x'_{st}\beta + U_{st} \tag{2}$$

In the first stage, we fit a standard Poisson log-linear model, i.e. omitting the random effects U_{st} in equation 2, to identify potentially important covariates. This is a conservative strategy in the sense that failure to take account of random effects typically leads to spuriously small standard errors of regression parameters.

In the second stage, we fit a full, mixed effects model including the covariates identified in stage one. We estimate the parameters β and θ by Bayesian inference. This requires us to specify prior distributions for the parameter vectors β and θ. In what follows, we specify independent priors for β and θ, and use Y and U as shorthand for the complete sets of values of Y_{st} and U_{st}, respectively. Writing $[\cdot]$ to denote "the distribution of" and a vertical bar to denote conditioning, this results in the following model structure,

$$[Y, U, \beta, \theta] = [Y|U, \beta, \theta][U|\theta][\beta][\theta] \tag{3}$$

For parameter estimation, we apply Bayes' theorem to obtain the joint posterior distribution of β,θ and the random effects U to give

$$[\beta, \theta, U|Y] = [Y, \beta, \theta, U] / \int [Y, \beta, \theta, U] d\beta d\theta dU \tag{4}$$

The integral in the denominator of 4 is analytically intractable, but we can sample from the joint posterior using Markov chain Monte Carlo (MCMC) methods; we used both Metropolis-Hastings and Gibbs sampling. We generated 3 chains of length 300,000 after a burn-in of 50,000 iterations, retaining every fiftieth iteration to obtain a sample of 5000 approximately independent realisation from the joint posterior distribution of β, θ and U for post-processing. To assess the convergence of the chains, we used visual inspection of trace plots and the Geweke statistic (Geweke et al., 1991) as well as the Gelman-Rubin diagnostic (Gelman, Rubin, et al., 1992). To carry out the Geweke test, a chain is divided into early and late windows containing the iterates. If the chain has achieved stationarity, the means of the values in the two windows are similar. The statistic is given by the difference between the two means divided by the standard error of their difference. The Z statistic is the test statistic and $Z \sim N(0, 1)$ if the chain has converged.

4 Predictive mapping

A standard approach to mapping results is to calculate point predictions of the relative risks, R_{st}, which is defined as;

$$\log(R_{st}) = x'_{st}\beta + U_{st} \tag{5}$$

see, for example (Best, Richardson, & Thomson, 2005). The minimum mean square point predictor of R_{st} (or of any other quantity of interest) is its conditional expectation given Y, i.e. its expectation under the posterior distribution, which we estimate as the observed mean over MCMC samples. A useful refinement is to map separately the conditional expectations for a multiplicative decomposition of R_{st} into the *explained risk*, $\exp(x'_{st}\beta)$ and the *unexplained risk* $\exp(U_{st})$, again estimated as the observed means over MCMC samples.

A useful way to convey uncertainty in predictions, especially in situations where relative risk thresholds are linked to policy interventions, is using probability exceedance maps (Diggle et al., 2007; Diggle, Moraga, Rowlingson, Taylor, et al., 2013; Diggle & Giorgi, 2015). In these, the mapped quantity is the posterior probability that θ_{st} exceeds a specified threshold; where predictions are precise, the mapped probabilities will be close to zero or one.

A third option is to map quantiles of the posterior distributions of R_{st}. For example, posterior medians are an alternative to posterior expectations as point predictors, whilst 2.5% and 97.5% quantiles would correspond to point-wise 95% credible intervals.

5 Model diagnostics

Figure S4 (top panel) shows the trace plot of the overall posterior median for the fitted values. The stationary behaviour of the trace indicates convergence of the chain. The lower panel shows the serial correlation among the fitted values. After carrying out the Ljung-Box test, a value of $p = 0.56$ is obtained. Therefore we fail to reject the null hypothesis of zero autocorrelations.

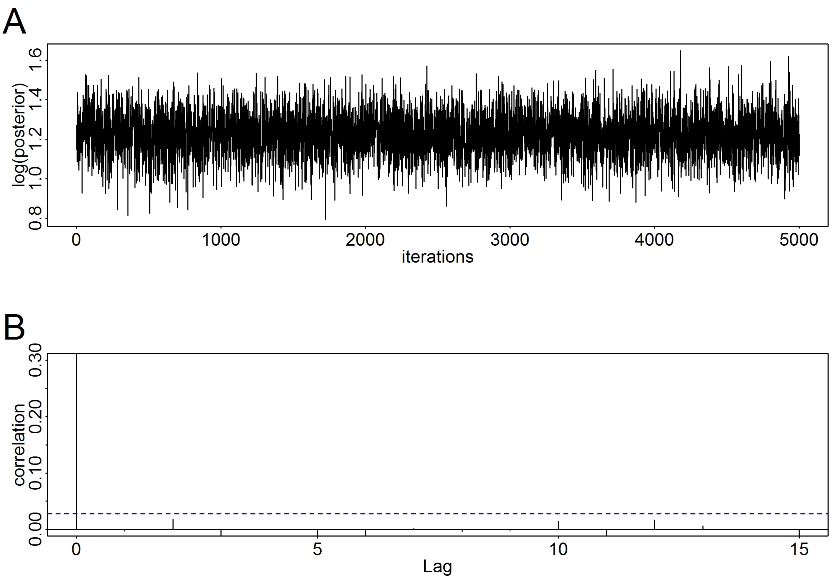

Figure S3: (A) Trace plot of the log of fitted values for 5000 samples from the model and (B) autocorrelation plot

6 Discussion

To check whether the included climate and non-climate variables fully explain the observed recent increase in malaria cases, we fitted an additional model with ITN in addition to the model in the main paper without the ITN. Results of observed malaria risk show comparable results indicating both factors do not explain away the increase in malaria. The figure below shows the total, unexplained and explained variation by the model covariates.

Table S1: Estimates for relative risk for climatic and non-climatic parameters respectively with associated 95% credible intervals

	RR	95% credible interval
Rainfall	1.00	(1.00,100)
Rainfall lag 1	1.00	(1.00,1.00)
Rainfall lag 2	1.00	(1.00,1.00)
Rainfall lag 3	1.02	(1.01,1.04)
Temperature	1.03	(1.00,1.05)
Temperature lag 1	1.04	(1.01,1.08)
Temperature lag 2	1.05	(1.04,1.08)
Temperature lag 3	1.04	(1.01,1.06)
NDVI	1.74	(1.57,2.06)
Literacy	1.00	(1.00, 1.00)
Pop. density	1.00	(1.00, 1.00)
RDT	1.26	(0.95, 1.67)
ITN	1.01	(0.97,1.02)

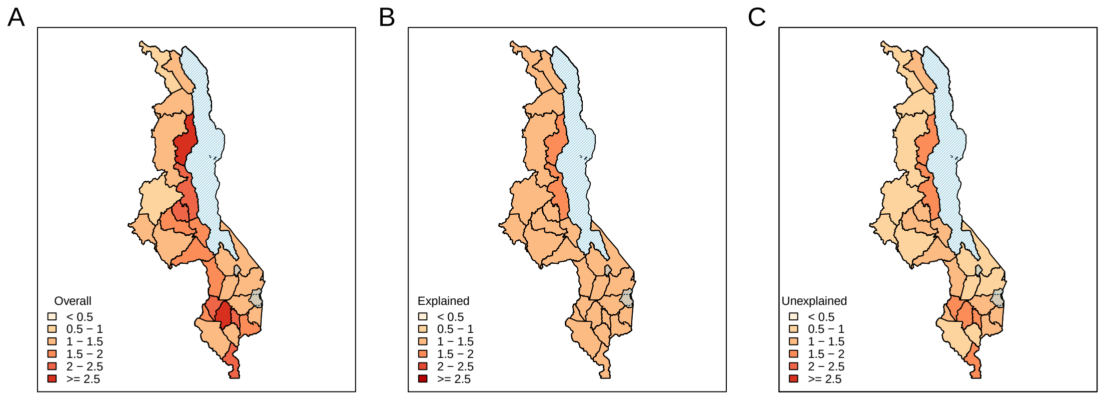

Figure S4: (A) Overall variation, (B)Explained and (C) Unexplained variation

References

Best, N., Richardson, S., & Thomson, A. (2005). A comparison of bayesian spatial models for disease mapping. *Statistical methods in medical research*, *14*(1), 35–59.

Cleveland, R. B., Cleveland, W. S., McRae, J. E., & Terpenning, I. (1990). Stl: A seasonal-trend decomposition procedure based on loess. *Journal of Official Statistics*, *6*(1), 3–73.

Diggle, P. J., & Giorgi, E. (2015). Model-based geostatistics for prevalence mapping in low-resource settings. *Journal of the American Statistical Association*(just-accepted), 1–42.

Diggle, P. J., Moraga, P., Rowlingson, B., Taylor, B. M., et al. (2013). Spatial and spatio-temporal log-gaussian cox processes: extending the geostatistical paradigm. *Statistical Science*, *28*(4), 542–563.

Diggle, P. J., Thomson, M. C., Christensen, O., Rowlingson, B., Obsomer, V., Gardon, J., ... others (2007). Spatial modelling and the prediction of loa loa risk: decision making under uncertainty. *Annals of Tropical Medicine & Parasitology*, *101*(6), 499–509.

Gelman, A., Rubin, D. B., et al. (1992). Inference from iterative simulation using multiple sequences. *Statistical science*, *7*(4), 457–472.

Geweke, J., et al. (1991). *Evaluating the accuracy of sampling-based approaches to the calculation of posterior moments* (Vol. 196). Federal Reserve Bank of Minneapolis, Research Department Minneapolis, MN, USA.

Ministry of Health (MOH). (2015). *Malawi health sector strategic plan 2011-2016.* Lilongwe, Malawi: MOH.
